# Production and applications of fluorobody from redox-engineered *Escherichia coli*

**DOI:** 10.1007/s00253-023-12395-6

**Published:** 2023-02-02

**Authors:** Witsanu Srila, Thae Thae Min, Thitima Sumphanapai, Kuntalee Rangnoi, Mehmet Berkmen, Montarop Yamabhai

**Affiliations:** 1 School of Biotechnology, Institute of Agricultural Technology, Suranaree University of Technology, Nakhon Ratchasima, 30000 Thailand; 2 New England Biolabs, Ipswich, MA 01938 USA

**Keywords:** Emerald green fluorescent protein (EmGFP), Single-chain variable fragment (scFv), Fusion, Fluorobody, Immunofluorescence, *E. coli* SHuffle

## Abstract

**Abstract:**

Efficient selection and production of antibody fragments in microbial systems remain to be a challenging process. To optimize microbial production of single-chain variable fragments (scFvs), we have chosen five model targets, 1) a hapten, Zearalenone (ZEN) mycotoxin, along with infectious agents 2) rabies virus, 3) *Propionibacterium acnes*, 4) *Pseudomonas aeruginosa*, and a cancer cell 5) acute myeloid leukemia cell line (HL-60). The scFv binders were affinity selected from a non-immunized human phage display scFv antibody library and genetically fused to the N-terminus of emerald green fluorescent protein (EmGFP). The scFv-EmGFP fusion constructs were subcloned into an expression vector, under the control of T7 promoter, C-terminally tagged with hexa-histidine and expressed in different *Escherichia coli* (*E. coli*) hosts. This enabled the detection of cells that expressed the correct scFv-EmGFP fusion, termed fluorobody, via bright fluorescent signal in the cytoplasm. Among the three *E. coli* hosts tested, an engineered *E. coli* B strain called SHuffle B that promotes disulfide bond formation in the cytoplasm appeared to be the most appropriate host. The recombinant fluorobodies were well expressed (2–8 mg/L), possessed the fluorescence property of EmGFP, and retained the ability to bind to their cognate targets. Their specific bindings were demonstrated by ELISA, fluorescence-linked immunosorbent assay (FLISA), flow cytometry, and fluorescent microscope imaging. The fluorobody expression platform in this study could be further adopted as a one-step immunostaining technique based on scFv, isolated from phage display library to numerous desired targets.

**Key points:**

• *E. coli SHuffle express T7 is a suitable expression host for scFv-EmGFP (fluorobody)*

• *Only the clones harboring scFv-EmGFP plasmid will show bright fluorescent signal*

• *This platform can be used to produce fluorobodies for numerous purposes*

**Graphical abstract:**

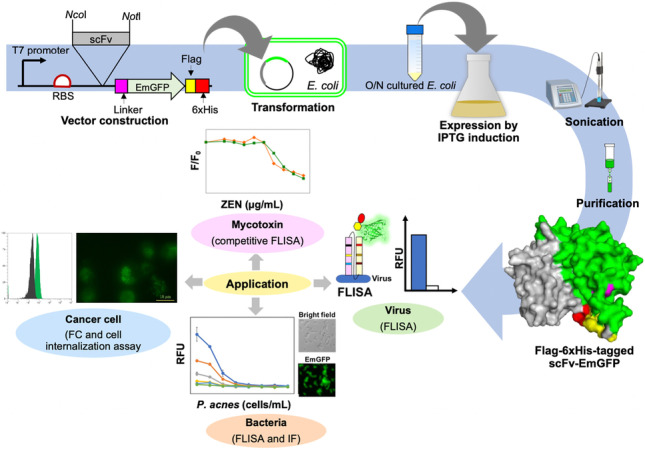

**Supplementary Information:**

The online version contains supplementary material available at 10.1007/s00253-023-12395-6.

## Introduction

Various types of antibody (Ab) fragments have been engineered for therapeutic and diagnostic purposes (Dübel 2007). The two common forms of small Ab fragments are single-chain variable-fragment (scFv) and single-domain Ab (sdAb). The scFv consists of light and heavy chain variable domains of immunoglobulin of mammals, linked with peptide linker (G_4_S)_3_. The average size of scFv is about 27 kDa and contains 2 predicted disulfide bonds (Ferrè and Clote 2005a). Phage display Ab technology is the most popular method for the generation of recombinant antibody (rAb) in the form of scFv or Fab. The key advantage of this technology relies on the simplicity but powerful affinity selection (bio-panning) procedure, and the direct linkage between the displayed Ab and its encoding gene within the phage genome (Kehoe and Kay 2005). Ab against desired target can be identified directly from diverse repertoires of Ab genes, generating high-affinity binding sites without the constraints imposed by classical method for generating either polyclonal or monoclonal Ab (McCafferty et al. 1990), such as high costs, stability of hybridoma cells, or batch-to-batch variation. Moreover, this method allows further engineering of the antibody into various formats to suit desired applications (Rangnoi et al. 2021), such as improved binding to FcγRI receptor (Robinson et al. 2015). Since the structure of the binding domain of the Ab is not glycosylated; contrary to therapeutic Ab which requires functional glycosylated constant domain (Fc), rAb can be expressed efficiently using expression systems which lack glycosylation machinery (Frenzel et al. 2013). This research focused on scFv format because it is small and can be obtained directly from biopanning of phage display scFv library. One of the common expression system for the production of scFvs especially for research purposes is *Escherichia coli* (*E. coli*), which is also a host for bacteriophage M13 (Spadiut et al. 2014). As expected, the expression level of various scFvs varied greatly, depending on different clones, despite a similar (65% identity) domain framework (Min and Yamabhai 2021).

For high sensitivity detection of biomolecules and the analysis of their interactions, Ab labeling with fluorescent probes, especially, fluorescein isothiocyanate (FITC) is one of the most widely used organic fluorophores in various applications (Hermanson 2008; Holmes and Lantz 2001). FITC can react with the free amino group of folded proteins, leading to stable thiourea bond and fluorescent Abs. However, the FITC may conjugate with the antigen-binding site of Abs, resulting in a partial or complete loss of reactivity of the Ab to its antigen (Sakamoto et al. 2012). To overcome these drawbacks, fusion of an *Aequorea victoria* green fluorescent protein (GFP) (Tsien 1998) with an scFv fragment, termed fluorobody, has been developed by genetic fusion of scFv to GFP. In this system, a direct conjugation of an scFv with a single GFP molecule (1:1 ratio) via a covalent bonding can improve the accuracy of quantitative analysis (Schwalbach et al. 2000). However, despite the use of fluorobodies as bio-probes in various detection formats, such as immunolabeling (Schwalbach et al. 2000), flow cytometry (Petrausch et al. 2007), fluorescent-linked immunosorbent assay (FLISA) (Oelschlaeger et al. 2002), the folding efficacy and the yield were generally poor (e.g., for fluorobody against s-triazine, 90% of the fusions were insoluble and only 25% could be refolded in *E. coli* BL21(DE3) pLysS) (Oelschlaeger et al. 2002). The yields of fluorobody against bacterial lipopolysaccharide (Griep et al. 1999) or hepatitis B surface antigen (HepBsAg) (Casey et al. 2000a) was ~ 100 to 200 µg/L in *E. coli* XL1-Blue-MRFX' Kan and Sure cells, respectively. This is likely because scFv has internal disulfide bonds (Ferrè and Clote 2005a), which require an environment containing enzymes that can promote the formation of correct bond formation between two cysteine molecules, such as the periplasm of *E. coli*. This requires the optimization of secretion of the scFv into the periplasm, where GFP is known not to fold (Ke et al. 2016). Further, the over-expression of protein in the periplasm to high yields in *E. coli* is known to be difficult to achieve (Karyolaimos and de Gier 2021; Olichon and Surrey 2007). To overcome these obstacles, *E. coli* SHuffle strains, which has been genetically engineered to promote correct disulfide bond formation in the cytoplasm (Lobstein et al. 2012), was selected as hosts for the expression of fluorobodies in this study. EmGFP (emerald GFP), which is a 27 kDa protein, derived from wildtype GFP (Ilagan et al. 2010) by introducing mutations (S65T, S72A, N149K, M153T, I167T), exhibits improved folding at 37 °C, is highly detectable, and has excitation maximum at 487 nm and emission maximum at 509 nm (Cubitt et al. 1999), was used as a fluorophore-fusion in this study. A suitable expression vector to produce scFv-EmGFP fusion or scFv-EmGFP fluorobody was also developed. The fluorobodies against diverse targets; namely, virus, mycotoxin, cancer cells and bacteria*,* were created and used as representative bio-detection probes with a myriad of applications in molecular biology. Finally, binding characteristic and their applications for quantitative and qualitative analysis in different assay formats were demonstrated.

## Materials and methods

### Materials, cell line, and bacterial strains

The pSERT/EmGFP vector containing EmGFP gene was purchased from Thermo Fisher Scientific (cat. #V35320, USA). Standard Zearalenone (ZEN) (cat. #CH-01S5) and ZEN conjugated with Bovine albumin serum; BSA (cat. #CJ-01-BSA) were prepared from Fusarium species (Aokin, Germany). Inactivated rabies virus (purified chick embryo cell vaccine, PCEC, LEP-Flury strain) was obtained from Rabipur (batch #2605, Chiron, India). *Propionibacterium acnes* DMST 14916 and *Pseudomonas aeruginosa* DMST 37186 were kindly provided by Dr. Griangsak Eumkeb, School of Sciences, Suranaree University of Technology, Thailand. *E. coli* SHuffle express T7 (*E. coli* SHuffle B, cat. #C3029, NEB, USA), SHuffle Express (*E. coli* SHuffle K-12, cat. #C3026, NEB, USA) and BL21 (DE3) (cat. #C2527, NEB, USA) strains were used to express fluorobodies. HL-60 cells were purchased from ATCC (cat. #CCL-240, Virginia, USA) and maintained with Iscove's Modified Dulbecco’s Medium (IMDM, cat. #12200036), supplemented with 20%FBS (cat. #10270106) and 1 × penicillin streptomycin (cat. #15140122). The cell culture media and supplements were purchased from Gibco (Eugene, OR, USA). Cells were cultured at 37 °C, 5% CO_2_.

### Construction of an expression vector to produce fluorobodies

**Fig. 1:**
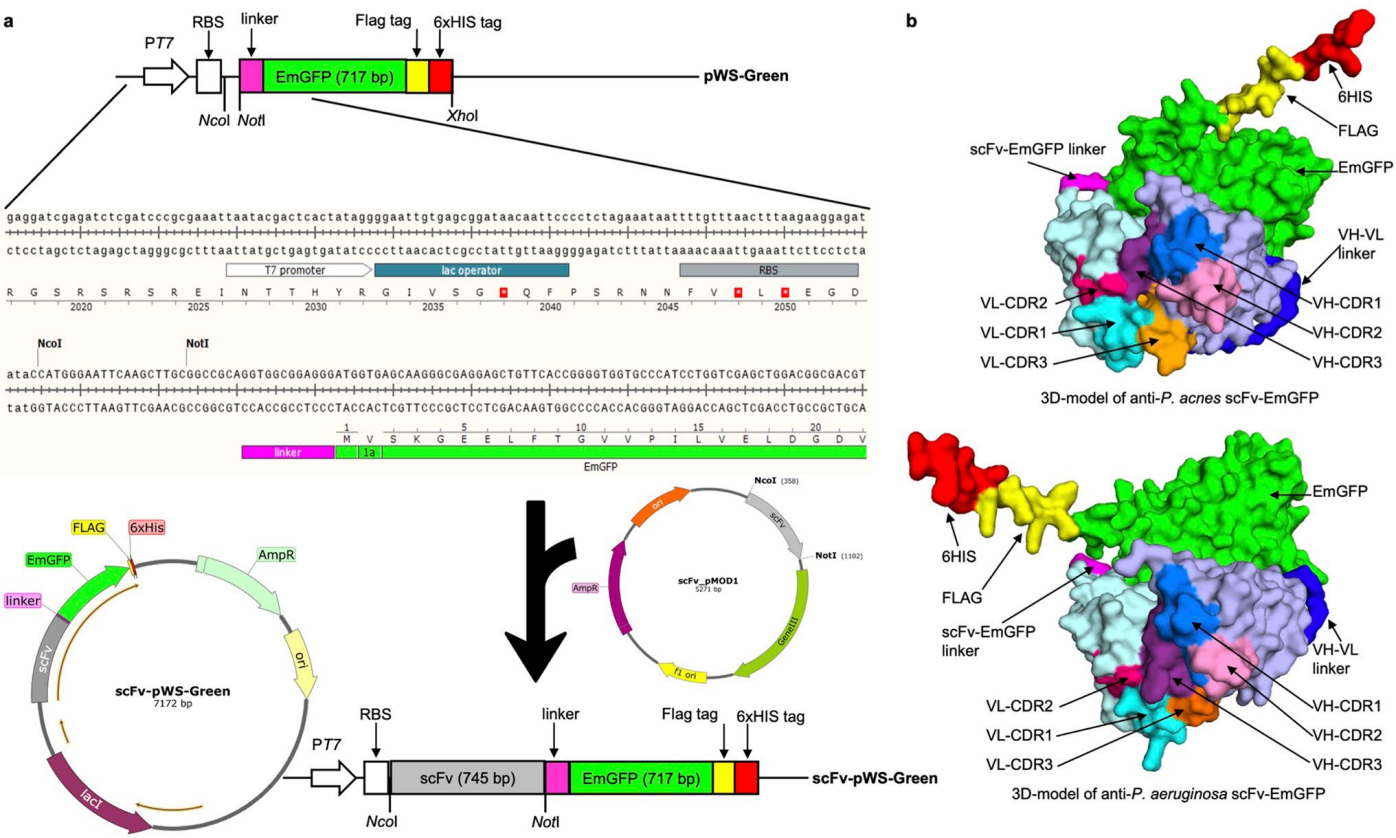
Construction of scFv-EmGFP fluorobodies. **A** Schematic representation and the DNA sequence of the regulatory 5′ UTR region of scFv-EmGFP constructs. The T7 promoter, lac operator, and ribosome binding site (RBS) along with the linker and EmGFP protein sequences are indicated. Amplified scFv DNA segments from phagemid vector (pMOD1) are digested and ligated into the *Nco*I and *Not*I restriction sites in the pWS-Green plasmid, resulting in fluorobody with 6xHis tag and FLAG tag at the C-terminus, to facilitate one-step affinity purification and detection. **B** 3D model of yPac1A8-EmGFP (anti-*P. acnes*) and yPgi3G4-EmGFP (anti-*P. aeruginosa*) generated by AlphaFold protein structure prediction and PyMOL molecular visualization system. The CDR regions of scFv, the VH-VL linker, scFv-EmGFP linker, EmGFP, 6xHis and FLAG tags are labeled

To construct a vector for the expression of scFv-EmGFP, the EmGFP gene from pSERT/EmGFP (cat. #V35320, Thermo Fisher Scientific, USA) was amplified by PCR using two primers; EmGFPNcoINotIFw: 5' CTG TGC CCA TGG GAA TTC AAG CTT GCG GCC GCA GGT GGC GGA GGG ATG GTG AGC AAG GGC GAG GAG-3' and EmGFPFlag6HisXhoIRv: 5'GCA CAG CTC GAG CTA GTG GTG GTG GTG GTG GTG CTT GTC GTC ATC GTC TTT GTA GTC CCC CTT GTA CAG CTC GTC CAT GCC-3', containing the *Nco*I and *Xho*I restriction sites (underlined), respectively. The amplified product was digested with *Nco*I and *Xho*I and ligated into the expression vector pET15b, which was digested using the same restriction enzymes. The map of the constructed plasmid, named pWS-Green is depicted in Fig. 1a. The integrity of the construct was confirmed by automated DNA sequencing (Macrogen, Korea).

### Cloning and expression of scFv-EmGFP fusions

To construct the scFv-EmGFP, the scFv genes, obtained from phagemid vectors (pMOD1) (Pansri et al. 2009), were digested with the *Nco*I and *Not*I restriction enzymes and ligated into corresponding digested pWS-Green (Fig. 1a). This resulted in the expression vector scFv-pWS-Green encoding scFv linked with N-terminus of EmGFP protein via G linker (GGGG) (Argos 1990), followed by C-terminal FLAG and 6xHis tags for one-step affinity purification and detection (Fig. 1a). The fused genes are under the control of T7 promoter and can be induced using isopropyl-β-D-thiogalactopyranoside (IPTG).

To produce the scFv-EmEGP fusions, the scFv-pWS-Green expression vectors were transformed into *E. coli* BL21 (DE3), SHuffle B and SHuffle K-12 strains to determine the suitable expression host. The fluorobodies were expressed according to previously published method (Lobstein et al. 2012) with some modifications. A single colony of each *E. coli* harboring the recombinant plasmid was inoculated into 5 mL of LB media containing 100 µg/mL of ampicillin and cultured at 30 °C with shaking 250 rpm, overnight. On the next day, 4 mL of overnight culture was inoculated into 400 mL of LB medium containing 100 µg/mL of ampicillin. Cells were cultured at 30 °C until OD_600_ reach 0.6, and expression was induced with 0.4 mM IPTG at 25 °C for 16 h before harvesting the cells. For negative control, the pET27b harboring gene encoding scFv fused with 6xHis was also expressed at the same time for comparison.

### 3D-modeling

Protein structure models of yPac1A8-EmGFP (anti-*P. acnes*) and yPgi3G4-EmGFP (anti-*P. aeruginosa*) were constructed by using AlphaFold Protein Structure Database (Jumper et al. 2021; Varadi et al. 2022) and PyMOL molecular visualization system from Schrödinger, LLC, USA (DeLano 2004).

### Purification of scFv-EmGFP fusions

*E. coli* cell pellets were harvested by centrifugation at 8,000 rpm for 10 min, then re-suspended in the binding buffer (20 mM Tris–HCl, 300 mM NaCl and 20 mM imidazole, pH 7.9) with 1 mg/mL lysozyme. Cells were disrupted by intermittent sonication at 25% amplitude for 7 min on ice using 30 s pulse and 30 s break for cooling after adding 1 mM phenylmethylsulfonyl fluoride (PMSF). The cell debris was removed by centrifugation at 15,000 × *g* for 20 min at 4 °C, and the clear supernatant was applied directly onto Ni–NTA column, pre-equilibrated with the binding buffer. After the column was washed with binding buffer, the fusion protein was eluted with elution buffer (20 mM Tris–HCl, 300 mM NaCl and 250 mM imidazole, pH 7.9). Fractions containing scFv-EmGFP fusion were pooled and exchanged by dialysis with PBS buffer and stored at 4 °C for analysis. The soluble fraction and purity of the samples were assessed by denaturing in sodium dodecyl sulfate–polyacrylamide gel electrophoresis (SDS-PAGE). The concentration of purified proteins were determined by Bradford method (Bradford 1976), using BSA as a standard.

### Gel electrophoresis and western blot analysis

The purified scFv-EmGFP fusions were analyzed by 12% SDS-PAGE. Protein bands were visualized by staining with Coomassie brilliant blue R-250 (Bio-Rad Laboratories, USA) (Laemmli 1970). All Blue Prestained Protein Standards (10–250 kDa) (cat. #1610373, Bio-Rad Laboratories, USA) was used as a molecular weight marker. Western blot was analyzed according to the manufacturer’s protocol (cat. #1703930, Bio-Rad Laboratories, USA). The proteins were electroblotted onto PVDF membrane at 100 V for 1.5 h, at 4 °C. Subsequently, the membrane was blocked for 1 h with 2% (w/v) skim milk in 1 × PBS (MPBS), at 4 °C for overnight. The membrane was then washed 3 times with 1 × PBS by rocking at room temperature for 1 min each time. After incubation with HisProbe-HRP conjugate (1:5000, cat. #15165, ThermoFisher Sceintific, USA,) for 1 h, the membrane was washed again. Finally, the protein target was visualized using Amersham ECL Prime Western Blotting Detection Reagent (cat. #RPN2232, GE Healcare Life Sciences, UK). Image was analyzed by ChemiDoc XRS Gel Documentation System (cat. #1708265, Bio-Rad, USA).

### Conventional ELISA

ELISA experiments were performed as previously described for yrPCE1A7-EmGFP (Pruksametanan et al. 2012), and yZEN2A8-EmGFP (Sompunga et al. 2019).

### Fluorescence-linked immunosorbent assay (FLISA)

A black Nunc-Immuno 96 well plate (cat. #437111, Thermo Fisher Scientific, USA) was coated with 1 μg of ZEN-BSA in 1 × PBS (for yZEN2A8-EmGFP) or 0.1 IU of rabies virus vaccine in 100 mM NaHCO3, pH 8.5 (for yrPCE1A7-EmGFP). For anti-bacterial fluorobodies, tenfold dilutions of heat-inactivated bacteria, ranging from 1.6 × 10^9^ to 1.6 × 10^2^ cells/mL in PBS were coated onto a black 96-well microtiter plate and incubated overnight at 37 °C, 1% (w/v) BSA or 2℅ skim milk was used as a negative control. After incubation at 4 °C overnight, the plates were blocked with 2% (w/v) MPBS at room temperature for 1 h, followed by washing 3 times with PBS. Then, 50 µL of 4% (w/v) MPBS and 100 μL of scFv-EmGFPs were added into each well and incubated at room temperature for 1 h. The wells were then washed three times with PBS containing 0.05% (v/v) Tween 20 (PBST) and twice with PBS. Finally, 100 µL of PBS were added. The fluorescence intensity was measured by a fluorescent microplate reader (Varioskan LUX multimode microplate reader (cat. #VL0000D0, ThermoScientific, USA). The excitation and emission wavelengths were 478–484 and 560–509 nm, respectively.

### Competitive FLISA

To confirm the binding of anti-ZEN (hapten), yZEN2A8-EmGFP, competitive FLISA was performed as previously described for competitive ELISA (Rangnoi et al. 2011). The optimum concentration of different targets and scFv-EmGFP were determined by checkerboard titration as previously described (Sompunga et al. 2019). The IC_50_ values were estimated from the plot of inhibition curve as F/F_0_ vs concentration of ZEN at 50% F/F_0_. F and F_0_ were fluorescence intensities in wells with various amount of ZEN and zero, respectively.

### Direct immunofluorescence staining of bacteria with fluorobodies

The immunofluorescence staining of *P. acnes* and *P. aeruginosa* using yPac1A8-EmGFP (anti-*P. acnes*) and yPgi3G4-EmGFP (anti-*P. aeruginosa*) was carried out as previously described for scFv-6xHis tagged (Min and Yamabhai 2021).

### Flow cytometry analysis of HL-60 cancer cell using scFv-EmGFP

HL-60 cells were blocked with human IgG for 30 min at room temperature. Cells were then washed with 1xPBS containing 0.1% (w/v) BSA. After that, 0.1 μg/μL of anti-HL-60 cells fluorobody (y1HL63D6-EmGFP) was incubated with the cells for 1 h on ice. Then, the cells were washed by centrifugation at 330 × *g* for 5 min, 4 °C and resuspended with 1xPBS containing 0.1% (w/v) BSA. Propidium iodide (1 mg/mL) at 1:1000 dilution was added to exclude dead cells. Fluorescent intensity was detected by flow cytometer (cat. #A24858, Attune NxT Flow Cytometer, Thermo Fisher Scientific, USA). The events were acquired up to 10,000 events from Propidium iodide (PI) (cat. #P3566, Thermo Fisher Scientific, USA) negative population. For cells that were stained with y1HL63D6 scFv, the cells were stained with DyLight® 488 Anti-6X His tag® Ab (cat. #ab117512, Abcam, UK) for 1 h on ice prior to the washing step.

### Cancer cell internalization assay

HL-60 cells were incubated with y1HL63D6-EmGFP at 37 °C, 5% CO_2_ for 2 h. Then, cells were striped with 0.05% (w/v) trypsin for 10 min before complete media was added and washed with PBS. Then, cells were carefully dropped onto a glass slide. After that, slow fade gold mountant (cat. #S36936, Invitrogen, USA) was added to mount the slide and prevent fluorescent signal fading. Cells were observed under fluorescence microscope (BX50 microscope (cat. #BX50-FL-PA, Olympus, Tokyo, Japan).

## Results

### Construction of an expression vector for fluorobody (scFv-EmGFP) production

Five scFv genes against ZEN (yZEN2A8) (Sompunga et al. 2019), rabies virus (yrPCE1A7: GenBank accession number OQ129413), *P. acnes* (yPac1A8) (Min and Yamabhai 2021), *P. aeruginosa* (yPgi3G4) (Min and Yamabhai 2021) and HL-60 AML cell line (y1HL63D6) (Sumphanapai et al. 2022), were obtained by bio-panning (affinity selection) of a compact non-immunized phage-display human scFv library named YamoI, which was constructed in our laboratory (Pansri et al. 2009). The scFv genes were cut from phagemid pMOD-1 vector that was used to construct the library, and subcloned into *Nco*I and *Not*I restriction sites of pWS-Green expression vector, such that scFv DNA fragment is fused with 5′ end of the EmGFP, resulting in the expression of scFv-EmGFP fluorobody. The construct is fused with FLAG tag, followed by 6xHistidine tag at the C-terminus for further detection and one-step purification (Fig. 1a). An example of the three-dimensional models of anti-bacterial scFv-EmGFP, namely, yPac1A8-EmGFP and yPgi3G4-EmGFP, is shown in Fig. 1b.

### Expression of scFv-EmGFP fusions from different *E. coli* strains

**Fig. 2:**
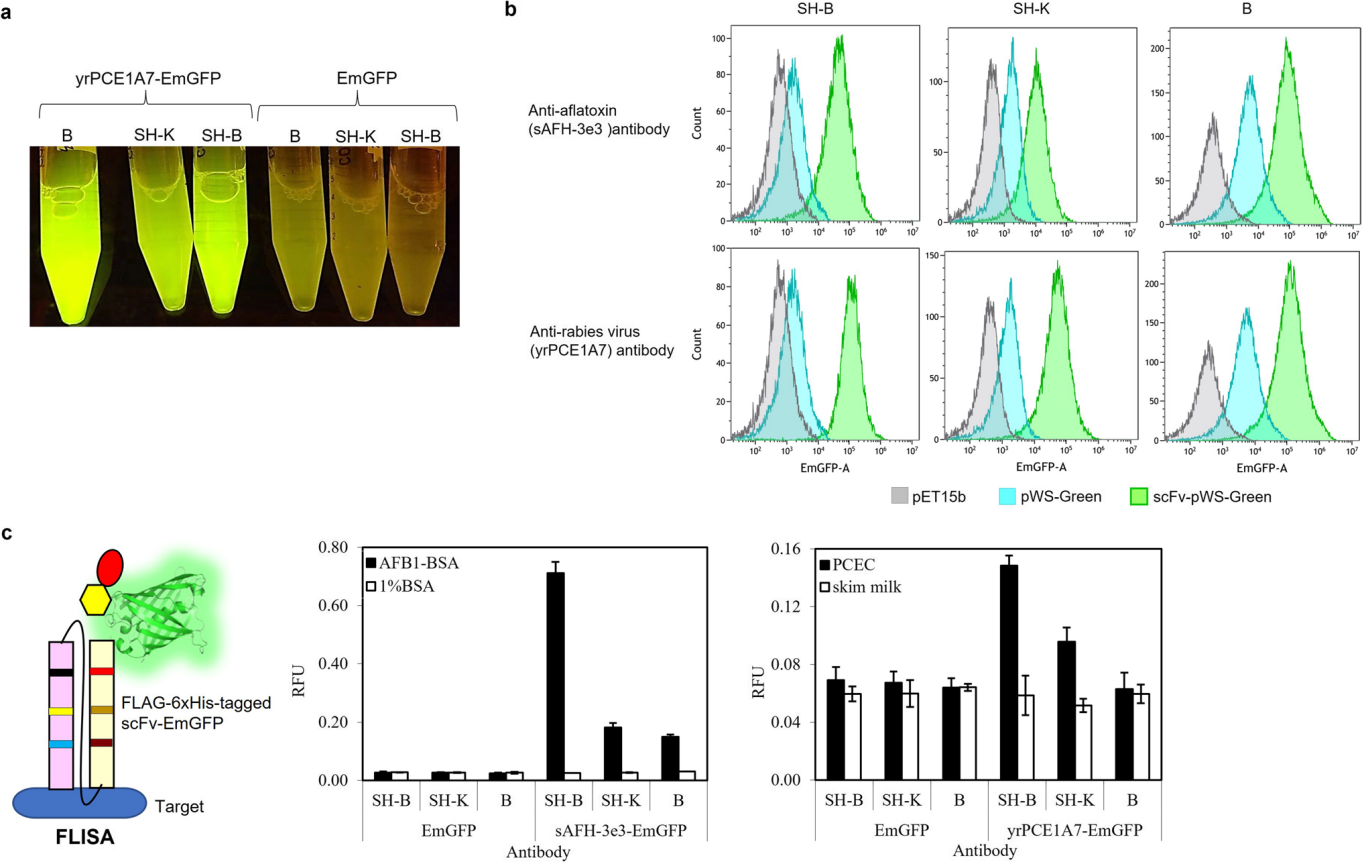
Properties of scFv-EmGFP generated from different *E. coli* expression hosts; wt *E. coli* BL21 (DE3), SHuffle K-12 and SHuffle B. **a** Tubes of cell lysate containing green fluorescence of scFv-EmGFP visualized with B-BOX Blue Light LED epi-illuminator (cat. #VE0100, SMOBiO, USA). **b** Flow cytometry analysis of three *E. coli* expression hosts (SH-B, SH-K and B) harboring 2 scFv-EmGFP constructs, yrPCE1A7- and sAFH-3e3-EmGFP. Empty pET15b vector was used as a negative control. **c** Schematic representation of how FLISA demonstrates the binding of scFv-EmGFP clones. EmGFP green beta barrel structure, blue antigen, scFv with linker, yellow hexagon FLAG tag and red oval 6xHis tag. sAFH-3e3- and yrPCE1A7-EmGFP obtained from crude cytoplasmic extract were checked for the binding activity against their targets by FLISA. BSA and skim milk were used as negative controls. The relative fluorescence units (RFU) values and standard errors from triplicate wells are shown. *E. coli* SHuffle B (SH-B); *E. coli* SHuffle K-12 (SH-K); wt *E. coli* BL21 (B)

In order to determine the suitable expression host for scFv-EmGFP expression, two scFv-EmGFP constructs, i.e., sAFH-3e3-EmGFP (Rangnoi et al. 2021) and yrPCE1A7-EmGFP were transformed into three different strains of *E. coli*, namely wild type (wt) DE3 and engineered SHuffle B and SHuffle K12 strains. The transformed *E. coli* appeared fluorescent green as shown in Fig. 2a. The level of fluorescence intensity was much higher for all *E. coli* strains expressing scFv-EmGFP when compared to EmGFP alone, as indicated by flow cytometry analysis (Fig. 2b). This is likely because for the empty vector, RBS is positioned distal from EmGFP start codon (43 bp) as illustrated in Fig. 1a. When scFv gene was subcloned between *Nco*I and *Not*I, the length between RBS and start codon is reduced (6 bp) to be optimal for translation, resulting in high level expression of the scFv-EmGFP fusion, which could be observed from cell lysate as bright fluorescent green as shown in Fig. 2a. Next, the binding of scFv-EmGFP fusion expressed from 3 different strains were determined by FLISA and ELISA. Two, fluorobodies (sAFH-3e3-EmGFP and yrPCE1A7-EmGFP) were used as examples of this study. As demonstrated in Fig. 2c and Figure S1, fluorobodies from engineered *E. coli* SHuffle B strain exhibited the highest binding activity among the three expression hosts when compared with engineered *E. coli* SHuffle K12 and DE3. Subsequently, all scFv-EmGFPs constructs were expressed in *E. coli* SHuffle B strain which has been genetically engineered to promote the formation for disulfide bonded proteins in the cytoplasm (Lobstein et al. 2012).

### Purification of scFv-EmGFP fusions

**Fig. 3:**
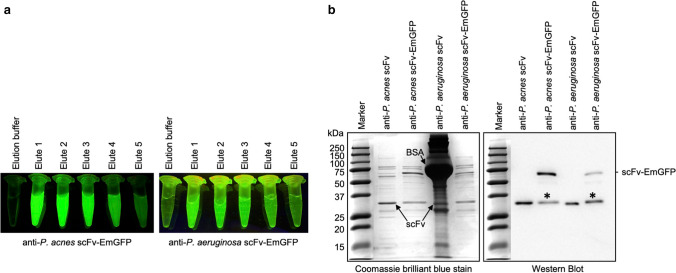
Purification of scFv-EmGFP fusion. **a** Fluorescence of eluted fractions of anti-*P. acnes* (yPac1A8-EmGFP) and anti-*P. aeruginosa* (yPgi3G4-EmGFP) are visualized by illuminating with B-BOX Blue Light LED epi-illuminator at 470 nm. The elution buffer alone was used as a negative control. **b** Reducing SDS-PAGE and western blot analysis of anti-bacterial fluorobodies. About 1.0 µg of anti-*P. acnes* yPac1A8 scFv (27.8 kDa), anti-*P. acnes* yPac1A8-EmGFP (56 kDa), anti-*P. aeruginosa* yPgi3G4 scFv (27 kDa, kept in 25.0 mg/mL BSA (arrowed); hence another big dark band), and anti-*P. aeruginosa* yPgi3G4-EmGFP (55 kDa) are loaded into each lane. All Blue Prestained Protein Standards is used as a marker. Asterisks are EmGFP fused with FLAG and 6xHis tags (28.8 kDa)

After IPTG induction, the fluorobodies were mainly expressed as soluble proteins in the cytosol of *E. coli* cells. To validate the correct active expression of fluorobodies, we purified them by one-step purification using Ni^2+^ affinity chromatography to determine the purity and size of the constructs by western blot and evaluate their binding activities. The purified products could be directly observed on a B-BOX Blue Light LED epi-illuminator as shown in Fig. 3a. The visualization of emerald-green fluorescence by fluorobody confirms the presence of the fusions in eluted fractions. SDS-PAGE and western blot analysis of partially purified products indicate that the anti-*P. acnes* yPac1A8-EmGFP (56 kDa) and anti-*P. aeruginosa* yPgi3G4-EmGFP (55 kDa) are soluble and expressed at the expected sizes (Fig. 3b). The parental anti-*P. acnes* yPac1A8 scFv (27.8 kDa) and anti-*P. aeruginosa* yPgi3G4 scFv (27 kDa) having 6xHis tags are used as controls. Western blot analysis using HisProbe-HRP revealed an extra band of nearly the same size as EmGFP fused with FLAG and 6xHis tags, in addition to the scFv-EmGFP band (Fig. 3b). Similar results were observed for other scFv-EmGFP as well (Figure S2 and S3). Besides, an extra band, which is likely to be an EmGFP dimer, was detected by western blot analysis of yrPCE1A7-EmGFP (Figure S3). These results suggested that proteolysis of the scFv-EmGFP occurred in *E. coli* (Casey et al. 2000a).

### Detection of mycotoxins

**Fig. 4:**
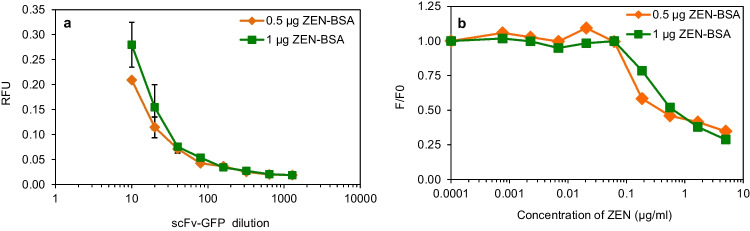
Detection of mycotoxin based on binding activity of yZEN2A8-EmGFP to ZEN by competitive FLISA. **a** Checkerboard titration FLISA of purified scFv-EmGFP dilution from 1:20 to 1:1,280 was performed against 0.5 µg/well of ZEN-BSA (orange line) and 1 µg/well of ZEN-BSA (green line). The data is expressed as absorbance at excitation peak at 487 nm and an emission peak at 506 nm. **b** Competitive FLISA to detect free ZEN by using 1:20 dilution of yZEN2A8-EmGFP against 0.5 µg/well of ZEN-BSA (orange line) and 1 µg/well of ZEN-BSA (green line) are illustrated. Absorbance values (expressed as F/F_0_) were plotted against the logarithm of free toxins concentration. The IC_50_ of yZEN2A8-EmGFP with different coating target are 0.3–0.55 µg/mL

To demonstrate the application of yZEN2A8-EmGFP for quantification of mycotoxin, competitive FLISA was performed. Firstly, optimal concentration of target and yZEN2A8-EmGFP protein were determined by checkerboard titration (Fig. 4a). Competitive FLISA was performed by using of 1:20 dilutions of yZEN2A8-EmGFP against 0.5 and 1.0 µg/well of ZEN-BSA. The IC_50_ of this scFv-EmGFP was approximaly 0.30–0.55 µg/ml, depending on coated ZEN-BSA per well. The best of IC_50_ value using this fluorobody was 0.30 µg/ml with a linear range from 0.1 to 5.0 µg/ml. (Fig. 4b). The limit of detection (LOD) of of this scFv-EmGFP Ab was 0.10 µg/ml. These results demonstrated that the yZEN2A8-EmGFP is applicable for the detection of contaminated mycotoxins as it can detect ZEN in range of the maximum allowance of contaminated in general food in Thailand (Anukul et al. 2013; Srianujata 2011) and EU (Wang et al. 2022), which were set at 30–1000 and 50–400 µg/kg, respectively.

### Detection of bacteria

**Fig. 5:**
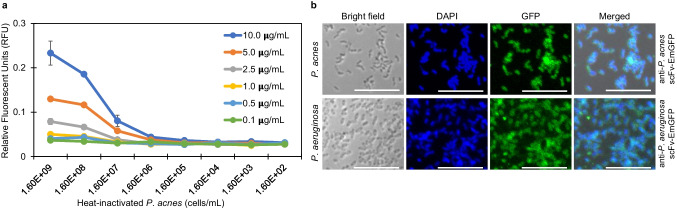
Detection of bacteria by scFv-EmGFP using **a** Utilizing FLISA, serial dilutions of *P. acnes* (from 1.6 × 10^9^ to 1.6 × 10^2^ cells/mL) are incubated with anti-*P. acnes* yPac1A8-EmGFP (from 10 to 0.1 μg/mL). 10 μg/mL of anti-*P. acnes* yPac1A8-EmGFP can detect 1.6 × 10^7^ *P. acnes*/mL. The lines represent the average value of duplicate samples and error bars represent the standard error of the mean. **b** Direct immunofluorescence staining of planktonic cells of *P. acnes* DMST 14916 (upper panel) and *P. aeruginosa* DMST 37186 (lower panel) by incubating with anti-*P. acnes* yPac1A8-EmGFP and anti-*P. aeruginosa* yPgi3G4-EmGFP, respectively, and counterstained with DAPI. In DAPI panel, nucleoids of bacteria are blue. In GFP panel, *P. acnes* cells are stained green by anti-*P. acnes* yPac1A8-EmGFP and *P. aeruginosa* cells are stained green by anti-*P. aeruginosa* yPgi3G4-EmGFP. In merged panel, the overlapping of blue nucleoids and GFP signals are seen. The scale bars represent 10 μm

To validate the application of fluorobody for the detection of bacteria, FLISA experiment was conducted on serial dilutions of *P. acnes* ranging from 1.6 × 10^9^ to 1.6 × 10^2^ cells/mL (Fig. 5a). *P. acnes* cells were incubated with various concentration of anti-*P. acnes* yPac1A8-EmGFP (0.1 to 10 μg/mL) and specific interaction was measured as fluorescence intensity in relative fluorescent units (RFU) by Varioskan LUX multimode microplate reader. FLISA result demonstrated that anti-*P. acnes* yPac1A8-EmGFP at 10 μg/mL could detect *P. acnes* at 1.6 × 10^7^ cells/mL. However, *P. acnes* cells could not be detected by yPac1A8-EmGFP at concentrations below 2.5 μg/mL. In conclusion, the yPac1A8-EmGFP could also be used to observe whole bacterial cells as shown by direct immunofluorescence staining in Fig. 5b.

### Detection of cancer cells

**Fig. 6:**
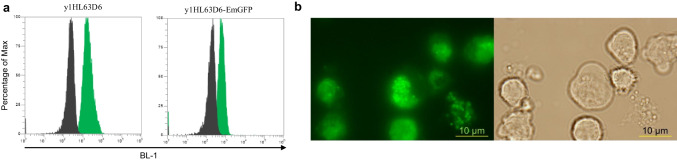
Application of scFv-EmGFP for cancer research. **a** Flow cytometry analysis of HL-60 cells against y1HL3D6 scFv and y1HL3D6-EmGFP. The fluorescent intensity was detected in BL-1 channel of the flow cytometer. Black peak: anti-His Dylight488 secondary Ab and PI, Green peak: y1HL3D6 scFv and y1HL3D6-EmGFP, respectively. **b** Image from fluorescent microscope showing in vitro endocytosis of y1HL3D6-EmGFP by HL-60 cells after incubation for 2 h. Cells were observed using GFP channel (left) and bright field channel (right). Endocytosed y1HL3D6-EmGFP are seen as green dots inside the cells

To investigate potential application of fluorobody for cancer research, scFv (designated y1HL63D6-EmGFP) against acute myeloid (AML) cell line (HL-60) which was selected by whole cell biopanning (Sumphanapai et al. 2022) was used as a model in this study. The binding ability of y1HL63D6-scFv-6xHis and y1HL63D6-EmGFP was compared by flow cytometry analysis. As demonstrated in Fig. 6a, the binding of y1HL63D6-EmGFP was slightly lower than that of y1HL63D6 scFv, with percentage of binding to HL-60 target cells at 95.0% and 99.5%, for y1HL63D6-EmGFP and y1HL63D6 scFv-6xHis, respectively. Next, potential application of y1HL63D6-EmGFP fluorobody for live cell imaging for in vitro internalization study was investigated. HL-60 cells were incubated with y1HL63D6-EmGFP at 37 °C for 2 h. The internalization of y1HL63D6-EmGFP was demonstrated by fluorescence microscope. Green-fluorescent spots appeared inside the cell outlines as shown in the bright field channel, indicating that after binding to the cell surface, the antibody could be internalized into the cells (Fig. 6b).

## Discussion

In order to demonstrate the board diversity of our non-immunized human phage display antibody library (Pansri et al. 2009), we selected and identified scFv binders to various targets, ranging from small molecule hapten to virus, bacteria and human cells (Min and Yamabhai 2021; Pansri et al. 2009; Pranomphon et al. 2018; Pruksametanan et al. 2012; Rangnoi et al. 2011, 2021; Sompunga et al. 2019; Vu et al. 2017). Functionality of the selected scFv binders fused to GFP as an in-built feature were validating using FLISA, flow cytometry and immunostaining, to determine their potential uses for various downstream applications. In this study, we described a rapid and simple cloning method to construct a fluorobody comprising of scFv linked to the N-terminus of EmGFP and express the resulting scFv-EmGFP fusion in the cytoplasm of an engineered *E. coli*. The cloning procedure facilitates subcloning of insert obtained from phage display vector (pMOD), directly into this scFv-EmGFP fusion plasmid (pWS-Green), without PCR amplification. Positioning a start codon too far from the ribosomal binding site in an empty EmGFP (43 nt) vector is the advantage of this expression system because only the clones harboring plasmids containing scFv insert will bring the start codon closer to the ribosomal binding site (6 nt), which is optimal for initiation of translation, resulting in a bright fluorescent signal in the cytoplasm of *E. coli*.

To discover the optimal *E. coli* strain for expression of scFv-EmGFP constructs, wt *E. coli* BL21 (DE3), redox engineered SHuffle K and SHuffle B strains were used to express various fluorobodies. SHuffle B strains lacking the two reductive pathways (Δ*trxB*, Δ*gor*) and expressing cytoplasmic disulfide bond isomerase (*dsbC*) was the ideal expression host, due to the disulfide bonds in scFv (Cys24/Cys98 and Cys160-162/Cys227-229), which are identified by DiANNA 1.1 web server (Ferrè and Clote 2005a, b, 2006)), required for correct folding, stability, and binding (Fass 2012; Huang et al. 2006; Wörn and Plückthun 1998; Zhao et al. 2010). However, the redox state of the whole scFv-EmGFP fusion, having 6 cysteines, remains to be characterized. Since the functionality of various fluorobodies have been demonstrated in this study, it is fair to conclude that any aberrant disulfide bond that may have formed in the scFv-EmGFP fusion, does not interfere with its functionality.

The position of GFP fusion relative to scFv appears to be critical for the functionality of fluorobody, i.e., fluorescent intensity and antigen binding properties. Previous reports have shown that GFP-scFv format was superior to scFv-GFP format when expressed in non-redox engineered *E. coli* strains. However, the GFP-scFv must be secreted into culture media (Liu et al. 2019) or periplasm (Casey et al. 2000b), or produced as inclusion bodies (Sakamoto et al. 2012). In this study, we show that 5 functional scFv-GFP could be efficiently produced as soluble fusion protein in the cytoplasm using redox-engineered *E. coli*. Therefore, it can be stated that functional fluorobody both in the form of scFv-GFP or GFP-scFv can be produced if appropriate *E. coli* host is used for fluorobody expression.

The Ni^2+^ affinity chromatography was not able to purify the fluorobodies to homogeneity as observed by SDS-PAGE (Fig. 3b) and previous reports (Griep et al. 1999; Oelschlaeger et al. 2002). However, we also demonstrated that the crude and partial purified fluorobodies could be used for ELISA, FLISA, competitive FLISA, flow cytometry, cell internalization assay and immunofluorescence staining. To the best of our knowledge, this is the first report on using yZEN2A8-EmGFP for Zearalenone detection by competitive FLISA immunoassay. The IC_50_ value was approximately threefold lower (higher sensitivity) than that of competitive ELISA using scFv (Sompunga et al. 2019). In addition to reduction of assay time, since competitive FLISA signal is not amplified by conjugated secondary Ab and the enzyme reaction, the accuracy of quantitative analysis could be enhanced (Jeong et al. 2014; Sakamoto et al. 2012).

Even if the limit of detection (LOD) of fluorobody against bacteria is higher than that of scFv-6xHis, the key advantage of using scFv-EmGFP fusion is being a one-step detection probe. Further optimization of the scFv-EmGFP construct such as fusion of GFP unit at N-terminal of scFv, optimization of the length of scFv-GFP linker or replace the EmGFP with a wide variety of mutated chromophore (Heim and Tsien 1996) might lead to improved detection sensitivity and applicable for detection of multiple strains.

Application of fluorobody for flow cytometry analysis has been previously reported (Petrausch et al. 2007). In their study, unlike our fluorobody produced in engineered *E. coli* expression system, the fluorobody was produced by yeast expression. Live cell imaging is useful for tracking cellular mechanism. The use of fluorobody allows a direct visualization of cells, avoiding the staining steps required for scFv detection. Here, we demonstrated that the y1HL63D6-EmGFP could be endocytosed into HL-60 target cells (Fig. 6b). Similar application of scFv fusion to C-terminus of GFP for cell-based analysis of LDL uptake mechanisms using GFP-scFv against LDL has been reported (Cardinale et al. 2004; Faulin et al. 2014).

Currently, blue, cyan, green, yellow, orange, red, far-red, and infra-red fluorescent proteins are available for biomolecular applications (Day and Davidson 2009). Data presented in this study demonstrated that fluorobody platform is a suitable format for many diagnostic applications which can be extended to fusions to other GFP variants, for multiplex assays.

In conclusion, this research shows that our bacterial expression platform using engineered *E. coli* SHuffle B strain is suitable to produce fluorobodies for the detection of a wide variety of targets.

## Supplementary Information

Below is the link to the electronic supplementary material.

Supplementary file1 (PDF 257 KB)

## Data Availability

The datasets generated and/or analyzed during the current study are available from the corresponding author on reasonable request.
