# Supplementary material for: Production and applications of fluorobody from redox-engineered Escherichia coli
Source: Appl Microbiol Biotechnol. 2023 Feb 2;107(5-6):1959–70. doi: 10.1007/s00253-023-12395-6 (PMC10050041; doi:10.1007/s00253-023-12395-6)
Supplement: Supplementary file 1 — Supplementary file1 (PDF 257 KB) [file 253_2023_12395_MOESM1_ESM.pdf]

Supplementary material

Applied Microbiology and Biotechnology

Production and applications of fluorobody from redox-engineered *Escherichia coli*

Witsanu Srila[1], Thae Thae Min[1], Thitima Sumphanapai[1], Kuntalee Rangnoi[1], Mehmet Berkmen[2] and Montarop Yamabhai [1*]

[1]School of Biotechnology, Institute of Agricultural Technology, Suranaree University of Technology, Nakhon Ratchasima 30000, Thailand.

[2]New England Biolabs, Ipswich, MA 01938, USA

[*] Corresponding author, email montarop@g.sut.ac.th; montarop@sut.ac.th

This file contains 3 supplementary figures

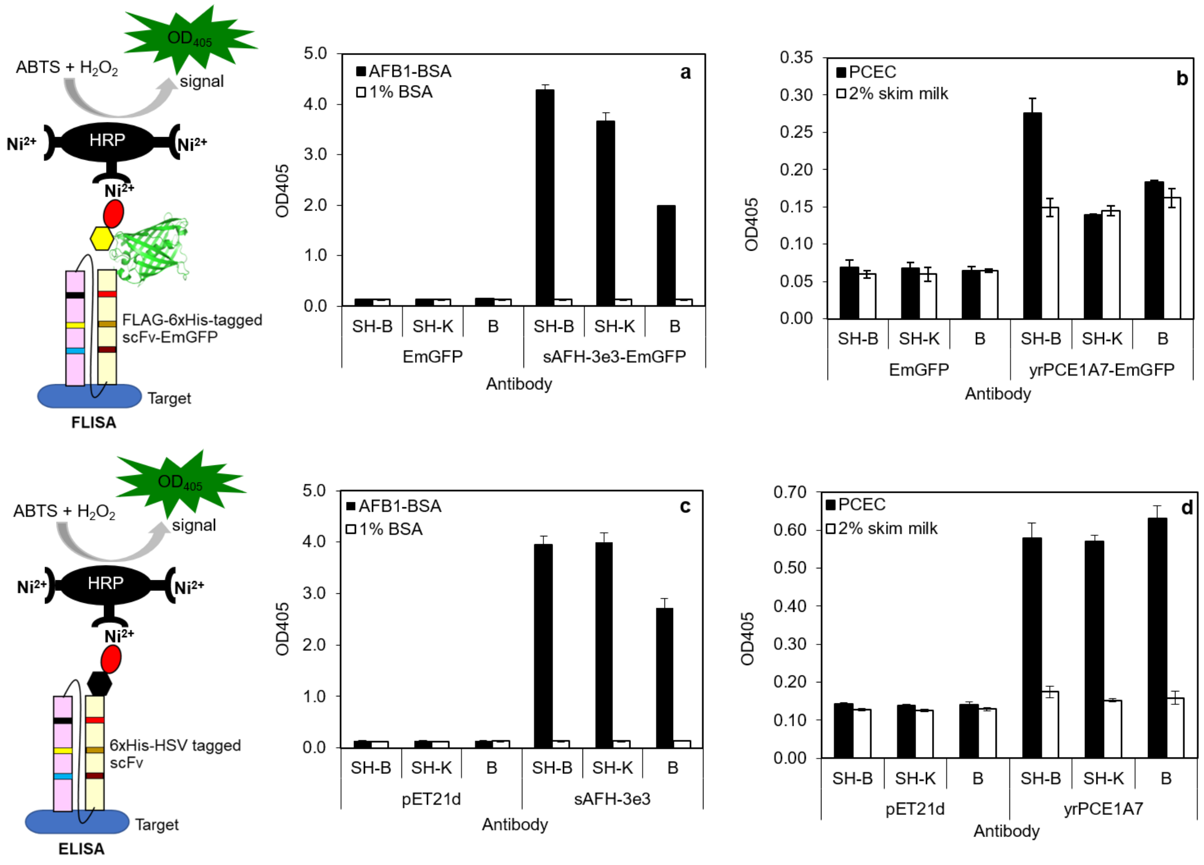

Figure S1 Binding property of fluorobody and scFv Abs. The scFv-EmGFP and scFv of sAFH-3e3 (**a** and **c**) and yrPCE1A7 (**b** and **d**) absorbance obtained from cytoplasmic space after induction and expression were determined by ELISA for binding to their target. BSA and skim milk were used as a negative control in this assay. The average optical density (OD) at 405 nm and standard errors from triplicate wells are shown. *E. coli* SHuffle B (SH-B); *E. coli* SHuffle K-12 (SH-K); wt *E. coli* BL21 (B).

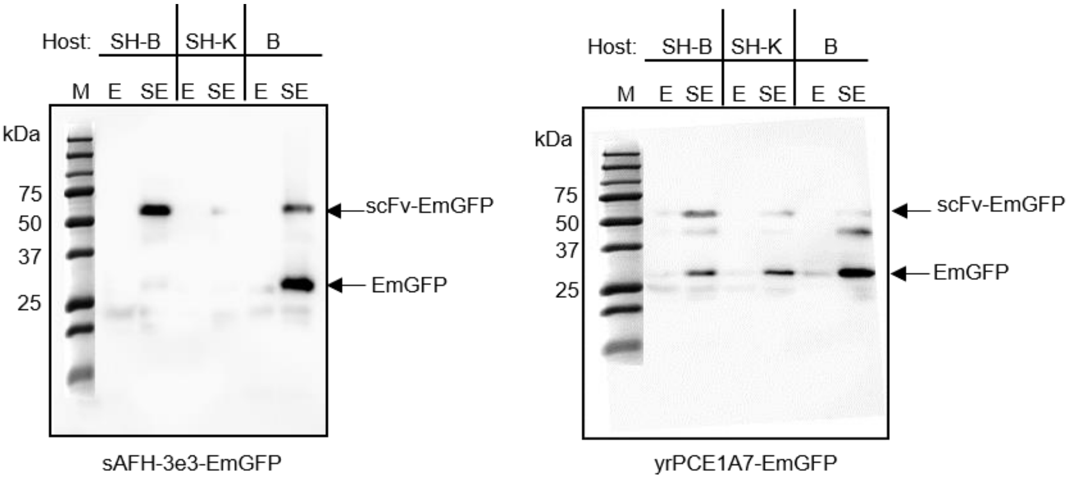

Figure S2 Western blot analysis of sAFH-3e3-EmGFP (55 kDa) and yrPCE1A7-EmGFP (55 kD) antibodies compared to the EmGFP (29 kDa) expressed from different *E. coli* hosts. Antibodies from cell lysate were transferred from 12% SDS-PAGE to PVDF membrane, were probed with HisProbe-HRP conjugate and visualized using Amersham ECL Prime Western Blotting Detection Reagent. Image analysis is performed by using ChemiDoc XRS Gel Documentation System (Bio-Rad, USA). The Precision plus Protein Standard was used as a protein marker (M). *E. coli* SHuffle B (SH-B); *E. coli* SHuffle K-12 (SH-K); wt *E. coli* BL21 (B); EmGFP (E); scFv-EmGFP (SE).

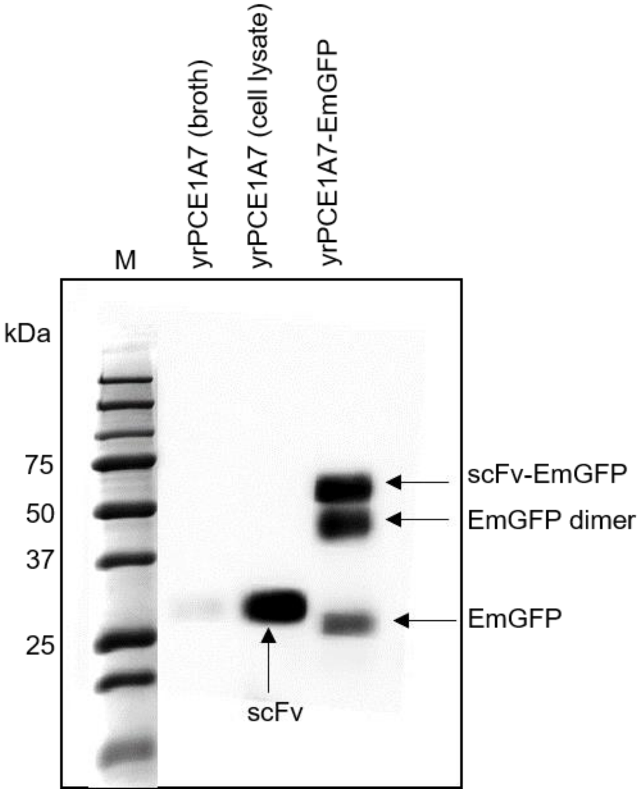

Figure S3 Western blot analysis of the purified scFv and scFv-EmGFP of yrPCE1A7 Ab. Abs expressed from *E. coli* SHuffle B cell lysate were treated with β-mercaptoethanol, transferred from 12% SDS-PAGE to PVDF membrane, probed with HisProbe-HRP conjugate and visualized using Amersham ECL Prime Western Blotting Detection Reagent. The Precision plus Protein Standard was used as a protein marker (M). The pET27b harboring yrPCE1A7 scFv gene was expressed in the same time for comparison. The molecular weight of yrPCE1A7 scFv antibody was estimated at 31 kDa. In a total lysate of the yrPCE1A7-EmGFP expressed from *E. coli* SHuffle B, molecular masses correspond to three products of protein were observed: a band with a molecular mass of approximately 55 kDa (scFv-EmGFP); a smaller band less than the 55 kDa for EmGFP dimer and non-fused EmGFP containing FLAG and 6xHis tags predicted to be 29 kDa.
